# Parasitological Assessment of Sewage Sludge Samples for Potential Agricultural Reuse in Tunisia

**DOI:** 10.3390/ijerph19031657

**Published:** 2022-01-31

**Authors:** Sonia Sabbahi, Layla Ben Ayed, Monia Trad, Ronny Berndtsson, Panagiotis Karanis

**Affiliations:** 1 LR16INRGREF02 Laboratoire de Recherche Valorisation des Eaux Non Conventionnelles, Institut National de Recherche en Génie Rural Eaux et Forêts (INRGREF) Rue Hédi Karray, Université de Carthage, Ariana 2080, Tunisia; rais.monia@iresa.agrinet.tn; 2 Laboratoire Sciences et Technologies des Eaux, Institut National Agronomique de Tunisie, 43 Avenue Charles Nicolle, Tunis 1082, Tunisia; benayedlayla@yahoo.fr; 3 Centre for Advanced Middle Eastern Studies, Division of Water Resources Engineering, Lund University, SE-22100 Lund, Sweden; 4 Department of Basic and Clinical Sciences, University of Nicosia Medical School, 21 Ilia Papakyriakou, 2414 Engomi, CY-1700 Nicosia, Cyprus; karanis.p@unic.ac.cy

**Keywords:** agricultural reuse, dried sewage sludge, helminth ova, protozoan cysts, semiarid climate, Tunisia

## Abstract

Wastewater sludge represents an important resource for reuse in agriculture. However, potentially harmful pathogens are a main threat in this context. Thus, the aim of this study was to examine the presence of helminth ova and protozoan cysts in dried sewage sludge samples collected from ten wastewater treatment plants (WWTPs) located in eight governorates in Tunisia. Based on morphological criteria, protozoan cysts of *Giardia* spp., *Entamoeba histolytica*/*dispar*/*moshkovskii*, and *Entamoeba coli*, were detected in all dried sludge composite samples (N = 116) from the investigated WWTPs. The mean concentration ranged from 1.4 to 10.7 cysts per 100 g dry matter (DM). The identified helminth eggs were *Ascaris* spp., *Strongyles*, Taeniid eggs, *Hymenolepis nana*, *Enterobius vermicularis*, and hookworm species. *Ascaris* spp. and Taeniid eggs were detected in 56.9 and 74.1% of analyzed samples, respectively. The presence of *Trichuris* spp., *Hymenolepis diminuta*, and *Toxocara* spp. eggs in dried sewage sludge samples was low (0.9, 1.7, and 2.6%, respectively). The mean concentration of helminth eggs during the three-year study was less than 1 egg/100 g DM. All examined dried sewage sludge sample contents were below the WHO (2006) and US EPA (2003) recommendations, and thus, the sludge can potentially be reused in agriculture.

## 1. Introduction

Production of sewage sludge is intrinsic to treatment of domestic wastewater, and its potential reuse as a fertilizer in agriculture provides a better alternative than incineration and landfilling. It has been estimated that 16 and 22% of all sludge/biosolids are either incinerated or landfilled, respectively, based on US EPA reporting data [1]. Agricultural use of sewage sludge has been recognized worldwide as a promising way to manage this resource, as it can minimize environmental pollution and contribute to circular economy from “waste to resources” [2]. The organic matter content in sludge can improve soil physical, chemical, and biological properties and produce favorable plant yield responses, when used as an organic fertilizer [3,4]. However, the main constraint remains the safety for reuse of the sewage sludge. There may be a risk in sludge reuse due to potentially concentrated harmful contents such as heavy metals, emergent pollutants, and pathogens.

In Tunisia, the annual production of the 110 operational wastewater treatment plants (WWTPs) of sewage sludge is approximately 35,100 metric tons of dry matter (DM) [5]. Agricultural reuse of sewage sludge has been carried out since 2008 as part of a pilot program conducted by the Ministry of Agriculture, aiming to improve the management system of: (a) monitoring and control of the safety and sustainable reuse of dried sewage sludge in agriculture and (b) to generalize its reuse throughout different governorates of the country. Under the national strategy, it is recommended to develop a program for use of the sludge, under maximum conditions of safety, health, and environmental protection. This could play a significant role in organic soil enrichment, while reducing sludge accumulation at wastewater treatment plants, landfills, and sources of serious environmental and health issues. This study is a part of the national product valuation strategy of the National Sanitation Utility (ONAS), and its 2006 action plan. In addition, the Sustainable Management of Sewage Sludge plan by the 2035 horizon, suggests developing scenarios encompassing each of the three sectors: agriculture (green sector), cement factories (red sector), and landfill (black sector), with the objective of ensuring sustainable management respecting the environment and public health. Landfilling does not entail disposal. Cement factories were proposed but have yet to be applied in practice. At present, the only remaining practical option is agricultural reuse, but this has still only been applied in a few cases. Furthermore, during the 2014–2015 agricultural seasons, reuse concerned only a portion of approximately 8% of the sludge produced by 12 treatment plants among the total 110 for a spreading quantity varying from 3500 to 3650 metric tons corresponding to spread areas of 307 and 335 ha for 2014 and 2015, respectively [5,6].

To control health risks regarding pathogens, several countries have developed legislation concerning the agricultural reuse of sewage sludge for the monitoring of microbial parameters especially helminth eggs. Protozoan parasites are generally excluded in countries’ legislation except for Russia. This is mainly related to the complex life cycle of helminths that differs from that of protozoan (oo) cysts. Moreover, their persistence in the environment remains much longer in soil and crops than for protozoan (oo) cysts [7]. As reported by Amoah et al. [8] based on the work of Pham-Duc et al. [9], hygiene is the main pre-disposing factor to an increased risk of parasitic infections (mainly protozoan) rather than exposure to wastewater or sludge. However, epidemiological studies highlight a high incidence of soil-transmitted helminth infections among farmers and consumers of vegetables irrigated with wastewater or sludge amended soil. According to Capizzi and Schwartzbrod [10], the establishment of a maximum concentration of viable eggs of helminths in sewage sludge is a worldwide criterion for agricultural reuse of sewage sludge. The recommended maximum contents of intestinal parasites in sewage sludge for agricultural reuse set by several countries are summarized in Table 1 [11,12,13,14,15,16,17,18,19,20,21,22,23,24,25,26,27,28].

In 2006, the World Health Organization suggested a viable helminth ova content limit of ≤1/g total solids (TS) in sludge [11]. In addition, the United States Environmental Protection Agency (US EPA) compliant with CFR part 503, defined treated solids as class A biosolids (use without site restrictions) when there is less than 1 viable helminth egg/4 g of TS (dry weight basis) and less than 10^3^ MPN (Most Probable Number)/g TS fecal coliforms [25]. The WHO guidelines and US EPA limits for helminth eggs, however, are based on limited epidemiological evidence, and on the performance of different sludge treatment methods [20]. They reported that it is necessary to correctly measure the helminth egg content in wastewater and sludge (treated or untreated) to enforce these limits. For other countries, no limit values concerning pathogenic helminth eggs in sludge reused in agriculture are set such as Greece, Portugal, Slovakia, Czech Republic, Denmark, Finland, Argentina, Italy, and Turkey [13,14,29,30,31]. To the authors’ knowledge, most Arabic countries such as Oman, Egypt, Iran, Lebanon, North Africa including Tunisia, Morocco, and Algeria have not determined limiting concentrations of helminth eggs in sewage sludge reused in agriculture [32,33,34,35,36]. The Tunisian standard NT 106.20 adopted in 2002 [32] has been applied for agricultural spreading of sludge. It specifies the requirements for use of WWTPs’ sludge as a fertilizer material in soil. The Tunisian standard NT 106.20 is extremely strict regarding heavy metals and fecal coliform content (<2 × 10^6^ MPN/g TS). However, it fails to consider the contents of pathogenic helminth eggs, which are of great concern due to their harmful effects on public health. Helminth eggs and pathogenic protozoa can be considered as the most difficult microbiological pathogens to inactivate in wastewater and sludge treatment [37]. Moreover, previous investigations of Tunisian treated wastewater showed the predominance of protozoan in sludge [38,39,40,41,42]. Indeed, Alouini [43] detected a dominance of protozoan (8.1·10^3^ cysts/100 g) over helminths (2.0·10^2^ ova/100 g) in sludge samples generated during primary and secondary wastewater treatment at the Charguia plant located in the greater district of Tunis, discovering *Giardia* spp. (4.3 to 5.1·10^3^ cysts/100 g) as the most prevalent parasite. According to Ben Ayed et al. [44], *Giardia* spp. was detected in dried and dehydrated sludge with a 25% presence (3/12). Local epidemiological data on diarrheic people [45] and stool samples of food handlers working in the Tunis greater area [46] have corroborated the predominance of protozoa prevalence over helminths. Thus, further investigation remains necessary.

According to Jiménez and Wang [47], not all reliable and affordable treatment processes in developing countries are efficiently adequate to inactivate eggs contained in sludge. Solar drying beds for sludge has become an economically feasible technique of sludge stabilization in the region, where warm and dry weather conditions are predominant [48].

In view of the above, the objective of the present study was to assess the feasibility of sludge reuse in agriculture based on its parasitological quality by analysis of samples at the laboratory scale. Due to a lack of limits in the Tunisian standard NT 106.20 regarding the agricultural reuse of sludge in Tunisia (no limit values are set for pathogenic microorganisms), results of the study can impact local decisions about sludge management according to the detected pathogenic microorganisms for a first assessment of potential health risks. The study was based on experimental and morphological assessment of the helminthic and protozoan pathogens on sludge samples dried by solar exposure in beds from ten WWTPs located in Tunisia and selected for first agricultural spreading trials of the dried sludge on demonstration pilot scale plots.

## 2. Materials and Methods

### 2.1. Study Area

**Figure 1:**
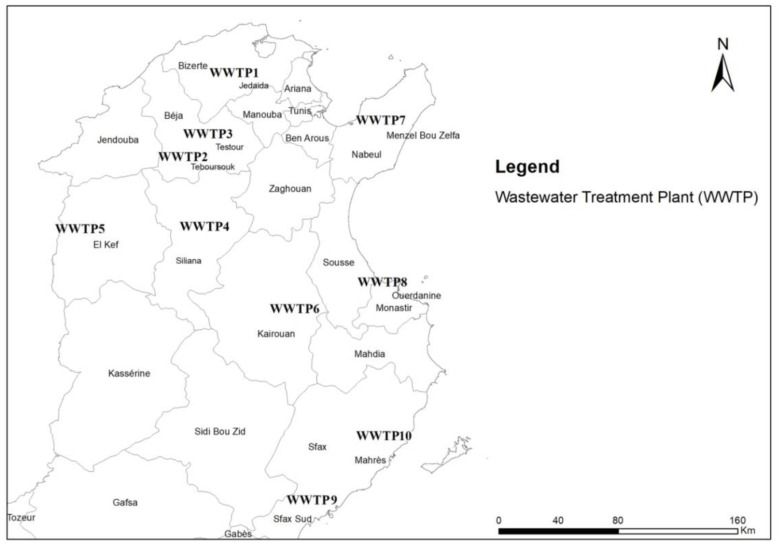
Location of investigated WWTPs with pilot practice for agricultural reuse of sludge [49].

The investigated WWTPs represent a major part of northern and central parts of Tunisia (Figure 1) using QGIS tool for mapping [49] with arid and semiarid climates. One WWTP is located in the greater district of Tunis (WWTP1), four of the plants are located in the northwest (WWTP 2-WWTP 5), one in the center (WWTP 6), two in the southeast (WWTP 7 and 10), and two in the eastern part of the country (WWTP 8 and 9). Geographic coordinates are shown for each of the WWTPs in Table 2.

### 2.2. Sludge Treatment and WWTP Characteristics

Table 2 shows the main operational process characteristics of each WWTP, namely capacity, flow rate, applied secondary wastewater treatment type, sludge volume, and main sludge treatment process. The ten WWTPs bear capacities for equivalent inhabitants ranging from 10,000 (WWTP 10) to 526,800 (WWTP 9) and treat sewage flow rates varying from 780 to 49,500 m^3^/day with an organic capacity ranging from 400 (WWTP 10) to 21,600 kg (WWTP 9) BOD_5_ per day. The WWTPs are characterized by two kinds of secondary biological treatment: activated sludge (WWTP 5) and oxidation dishes (WWTP 1–4 and 6–10). The 10 WWTPs efficiency is indicated by 80–97%, 71–94%, and 72–97% treatment efficiency for biological oxygen demand (BOD_5_), chemical oxygen demand (COD), and total suspended solids (TSS), respectively. The WWTPs were selected due to existing pilot scale agricultural reuse of the sludge according to Figure 1.

The sewage sludge treatment at the WWTPs includes thickening and dewatering in beds. Furthermore, among the ten WWTPs, six use aerobic sludge digestion simultaneously carried out in aeration tanks (WWTP 1–3, 5, 9, and 10) and the other four (WWTP 4, 6, 7, and 8) use stabilization through drying beds with solar exposure. However, periodically the aerobic digesters are not fully functional due to operational and technical problems. Stabilization of excess sludge is generally not sufficiently ensured due to deliberate limitation of aeration for energy saving purposes. Therefore, digestion of the sludge begins in the digesters and continues in the drying beds, and in the site storages of the WWTPs until reuse. To overcome this technical and economic limitation, aeration should either increase in the activation basins, or proceed to the digestion of the excess sludge (WWTPs 2 and 3). The drying beds are filled to capacity with layers varying in height approximately from 50 to 70 cm. The plants’ annual capacity of generated dried sludge ranges from 180 to 5813 m^3^/day for which dry matter content after drying is between 50 and 90% (Table 2). In Tunisia, lime treatment is not used after the dewatering process in WWTPs. Liming is carried out only during storage of the sludge at the plant sites, after extraction from drying beds or after mechanical dehydration especially in summer, and occasionally due to unpleasant odor problems or proliferation of mosquitoes.

### 2.3. Environmental Weather Conditions

**Figure 2:**
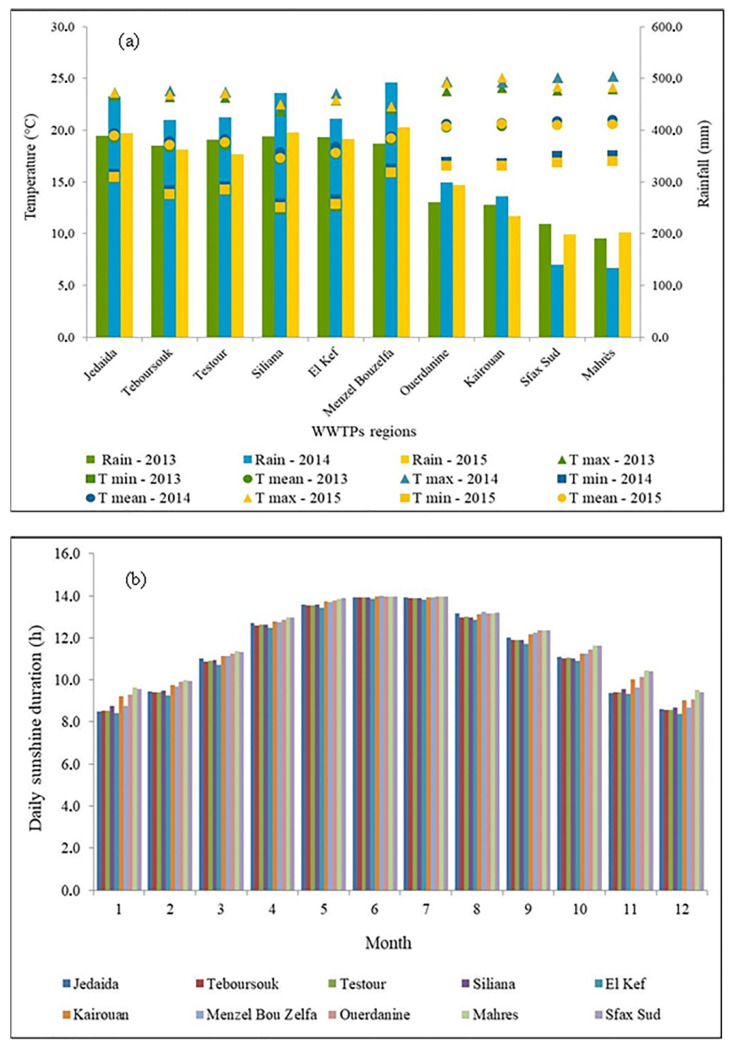
(**a**) Mean temperature, annual rainfall, and (**b**) sunshine duration distribution at the ten WWTPs during the study period (2013–2015).

According to Köppen’s climate classification, all study sites are classified as Csa characterized by a Mediterranean hot dry summer climate. In general, winters are mild with moderate rainfall and summers are hot and dry. Temperatures can exceed 40 °C in July and August. Temperature and rainfall data were retrieved from the ERA5 sites [50], and sunshine duration was retrieved from the ARA-interim site [51] (Figure 2a,b). The annual rainfall ranged between 134.3 and 492.3 mm during the three study years (2013–2015) for the southern and northern WWTP locations, respectively. The mean temperature ranged between 17.1 and 20.9 °C (Figure 2a). The country benefits from an average rate of sunshine of more than 3000 h/year. For the three-year study period, the mean insolation was 11.6 ± 0.2 h per day in the WWTP areas. Furthermore, there is a monthly variation in the sunshine duration with maximum in June and July (Figure 2b). On the contrary, minimum sunshine is recorded from November to February with more than 8.1 h per day.

Sewage sludge was exposed to sun in solar drying beds and sludge sampling was conducted throughout days without rainfall. Solar irradiation levels varied from a maximum of 894 W m^–2^ (full sunshine, summer) to a minimum of 500 W m^–2^ (winter to spring).

### 2.4. Sampling and Analyses

In total, 116 samples from the ten WWTPs were collected during the investigated three-year period corresponding approximately to an average of four samples per year and WWTP. The collected samples from the drying beds consisted of numerous subsamples obtained from various points in the bed that were combined into a total representative sample according to 40 CFR Part 503.8 standards [25,52]. Sampling was performed at each WWTP in accordance with US EPA [53] recommendations from January to December 2013–2015.

For each of the ten WWTPs, representative manual composite sub-samples were obtained over the length of the drying beds and, from a cross section of the bed after solar drying ranging from 15 to 21 days in summer and 21 to more than 30 days in winter, to encompass small-scale variation. A minimum of five samples per drying bed was stored in sterile plastic bags, kept chilled during transport, and analyzed within a 24–48-h period. Composite dried sludge sub-samples of 100 g were subject to parasitic analysis in triplicate, collected at various points of the plastic bag containing an initial composite sample of about 5 kg.

### 2.5. Identification and Counting of Helminth Ova and Protozoan Cysts

At present, there is no standard method for the determination of helminth eggs in sewage sludge [54]. Except for *Ascaris* ova, a standard test method was used for detecting, enumerating, and determining its viability in sludge by a flotation procedure using magnesium sulfate (specific gravity 1.20) [25]. We used the standardized flotation method with saturated sucrose solution of specific gravity 1.29 applied to identify both protozoan (oo) cysts and helminth ova [55]. It is important to mention that the flotation method is suitable for the recovery of intestinal parasites with low concentrations in dried sludge samples. However, this technique may have a limit of quantification, i.e., it can deform the helminth eggs and protozoan cysts due to the specific gravity of 1.29. Thus, to overcome this limitation, longer periods of contact time between the flotation solution and the eggs should be avoided. The dried sludge was sieved through a polyester mesh (300 µm) to remove large particles. First, a long mixing and homogenization step was performed by adding 100 g of dried sludge to 300 mL of sodium dodecyl sulfate detergent (SDS) (10%) to remove large floating debris, release the eggs from solids, and to completely solve the dried particles. The mixture was stirred from 5 to 10 min using six centrifuge tubes with a capacity of 50 mL each containing the suspension and then removed and concentrated by centrifugation at 1200 rpm for 3 min. The supernatant was discarded and sediments were washed three times by tap water to remove SDS detergent. Then, diluted Sheather’s sucrose solution, obtained by a 3/5 dilution factor of the concentrated Sheather’s sucrose solution, was added in an equal volume to sediment, mixed by vortexing until homogenization, and centrifuged again at 4200 rpm during 15 min. The supernatant was removed and replaced with concentrated Sheather’s sucrose solution (prepared by boiling 500 g of sucrose in 320 mL of distilled water, 2.5 g of phenol was added as a preservative; specific gravity of 1.29 measured using a hydrometer) added in an equal volume of the sediment (1200 rpm during 5 min). Eggs, larvae, and protozoan cysts in the upper layer of supernatant were transferred to a McMaster counting slide chamber especially elaborated for parasitological examinations and composed of two sub-chambers [54,56] filled with the suspension of dried sludge mixed with a given volume of the flotation solution using a Pasteur pipette. The full slides were left to stand on a flat surface for 5 to 10 min before examination so that the eggs would float on the surface. Finally, an average of three McMaster counting cells containing the amount of suspension was carefully examined under light microscopy with 10× or 40× magnification. Considering that three McMaster countings were taken per tube and that the third was usually negative, indicated that the entire meniscus of all centrifuge tubes of the same sample in which eggs and cysts are located was examined and counted. The result of counting is directly expressed per 100 g DM. Thus, all eggs were counted from the meniscus for all centrifuge tubes for each sample.

### 2.6. Statistical Analyses

Statistical analyses were performed using the R software environment for data analysis and graphics (R Version 3.3.0, 2016) [57]. Normality of data was verified by using the Pearson correlation coefficient. The data did not follow a normal distribution hence a non-parametric method was used for analysis. The non-parametric models used for statistical analysis included descriptive statistics and, univariate and bivariate analysis. The arithmetic mean of the three-year study period was used to present results for each parasite. The mean number of parasitic organisms per 100 g dried sludge was determined during the study period and the total data were statistically analyzed with quantitative descriptive statistics.

## 3. Results and Discussion

### 3.1. General Parasites (Ova and Cysts) Distribution in Sludge Samples

**Figure 3:**
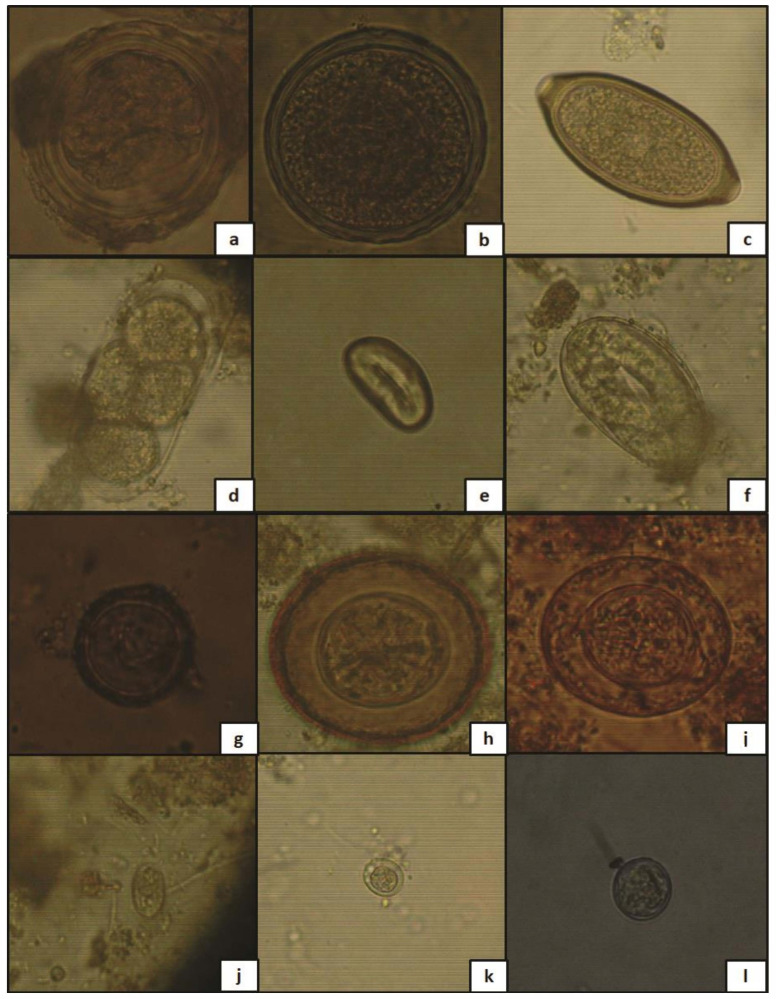
Light microscopic isolated images of common helminth ova found in sludge samples (magnification of 400x): (**a**) *Ascaris* spp., (**b**) *Trichuris* spp., (**c**) *Toxocara* spp., (**d**) Hookworms (*Ancylostoma duodenale* and *Necator americanus*), (**e**) *Enterobius vermicularis*, (**f**) *Strongyloides stercoralis*, (**g**) Taeniid eggs, (**h**) *Hymenolepis nana*, (**i**) *Hymenolepis diminuta*; (**j**) *Giardia* sp., (**k**) *Entameoba histolytica*/*dispar*/*moshkovskii*, and (**l**) *Entamoeba coli*.

**Figure 4:**
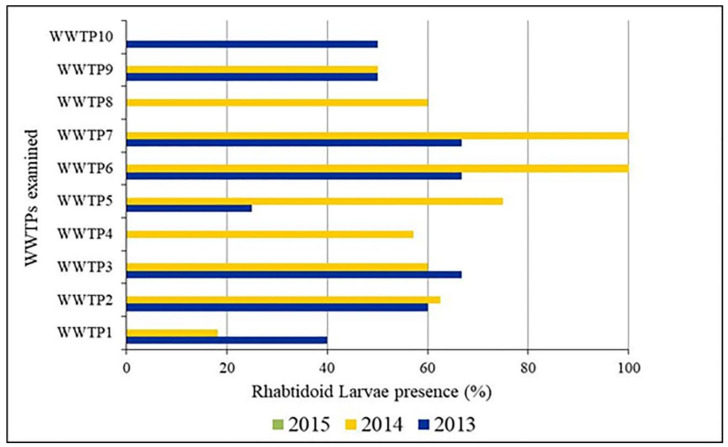
Percent presence of rhabditoid larvae of nematodes identified in dried sludge samples.

**Figure 5:**
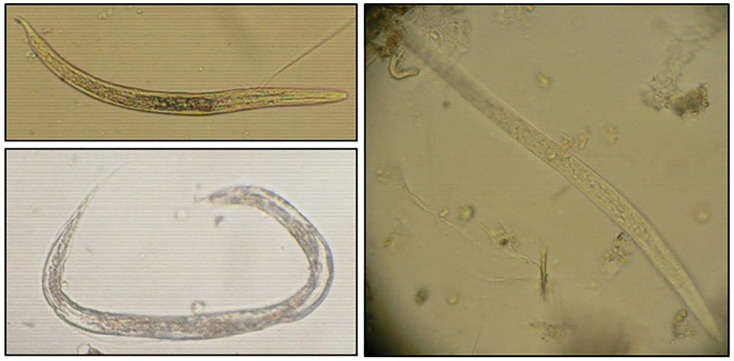
Light microscopic-isolated photos of rhabditoid larvae of nematodes identified in dried sludge samples.

Fourteen different common enteric parasites genera were present in the investigated sludge including helminth (nematodes and cestodes) ova and protozoan cysts (Figure 3 and Table 3). Nematode eggs belonged to *Strongyloides* spp. (91.4%) (106/116), *Strongyloides stercoralis* (*S. stercoralis*) (72.4%) (84/116), *Ascaris* spp. (56.9%) (66/116), *Tristrongyloides* spp. (44.0%) (51/116), *Enterobius vermicularis* (*E. vermicularis*) (37.9%) (44/116), hookworm species (*Ancylostoma duodenale* and *Necator americanus*) (28.4%) (44/116), *Toxocara* spp. (2.6%) (3/116), and *Trichuris* spp. (0.9%) (1/116). Among cestodes, Taeniid eggs were present in 74.1% (86/116), *Hymenolepis nana* with 67.2% (78/116), and *H. diminuta* only in 1.7% (2/116) of samples (Table 3). Protozoan cysts of *Giardia* spp., *Entamoeba histolytica*/*dispar*/*moshkovskii* and *Entamoeba coli* were detected in all samples (100%). By using light microscopy, the presence/ absence of larvae, was additionally considered. Rhabditoid larvae were found in 2013 (varying from 0 to 66.7%) and 2014 (from 0 to 100%), but not in 2015. It is, however, difficult to morphologically distinguish individual species of some parasitic helminths whose larvae were observed (*Strongyloides* spp., *Rhabditis* spp., *Tristrongylus* spp., and Hookworms) (Figure 4 and Figure 5). In laboratory and field diagnosis of helminths, larvae count, and identification are important. But few studies have taken into consideration their recovery from environmental matrices such as sludge. It is not evident, however, to explain the present results as in general the inhibition of larvae may be related mainly to seasonal conditions (moisture rate, dry season, high temperature) which was not the case in our study due to similarities of climatic conditions between 2013, 2014, and 2015, and type of larvae recovered according to the used methodology. Based on the literature, hookworm larvae may be recovered using the Baermann method [58,59]. We hypothesize that sludge samples taken during 2015 could as well have contained hookworm larvae. However, these are not easily detected by the saturated sucrose method used. Indeed, further studies are needed, and in our future investigations we will further consider, and identify the presence of larvae by employing several techniques.

Descriptive statistics of helminth eggs and protozoan cyst concentrations detected in the dried sludge samples, from all considered WWTPs and the entire study period (N = 116 samples) are shown in Table 3. Firstly, representative subsamples were separately analyzed for each of the ten WWTPs, and for each year separately. However, no significant differences were found between the 10 WWTPs investigated, and no seasonal variation was noted during the study period. Due to the similarity of results for all study years and all examined WWTPs, only one set of data is presented here (Pearson correlation coefficient, *p* > 0.05). Descriptive statistics show that mean contents of the quantified parasites (nematode and cestode eggs) are extremely low and that the coefficient of variation, especially for mean number per 100 g DM, indicates a high degree of uniformity of the collected samples for most quantified species (*Ascaris* spp. and *E. vermicularis*; Taeniid eggs, *H. nana*, and *S. stercoralis*) (Table 3). The parasitic examination included detection of especially genera *Ascaris* spp., *Trichuris* spp., and *Toxocara* spp. eggs. The latter two were not detected in the majority of the dried sludge samples compared to eggs of other detected species. The concentration of *Toxocara* spp. and *Trichuris* spp. eggs ranged between 0 and 0.3 eggs with a mean value close to zero, while more than half of the samples were contaminated with *Ascaris* spp. eggs with a mean of 0.3 ± 0.3/100 g DM (minimum 0 and maximum 1.8). Numerous authors and European regulations have argued that *Ascaris* spp., *Trichuris* spp., and *Toxocara* spp. eggs can be used as indicator parasites for control and evaluation of the efficiency of methods used for treatment of sewage sludge mainly due to their pathogenicity, occurrence, long survival, and resistance to environmental physicochemical factors, in addition to their higher specific gravity resulting in rapid settling (e.g., [60,61,62]). The concentration was below the limit values set by international legislations such as US EPA [25] and WHO [11] for three of the investigated Tunisian WWTPs. Moreover, it is important to determine their viability in sewage sludge for true parasitological safety assessment of dried sludge.

**Figure 6:**
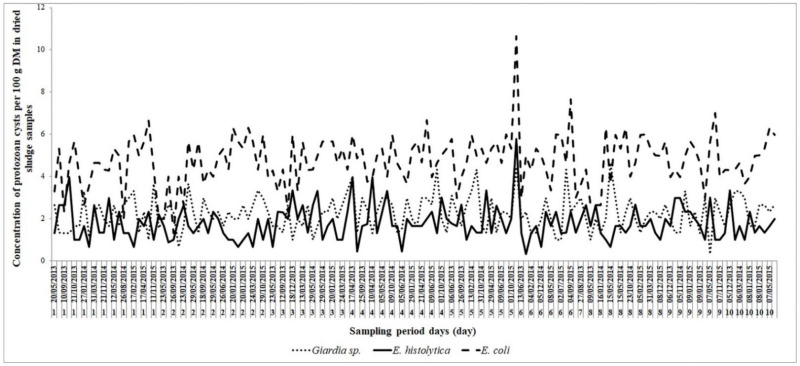
Protozoan cyst concentration (per 100 g DM) by Arther’s technique in dried sludge dewatered in beds at solar exposure from the ten WWTPs (1–10) during 2013–2015. Mean helminth egg concentration during the three-year study for all WWTPs was ≤1 egg/100 g DM.

During the study period, small fluctuations in the concentration of all helminth eggs were observed in the samples collected from the drying beds (Figure 6). Moreover, the mean helminth egg concentration during the three years of study for all investigated WWTPs was low and in the range of ≤1 egg/100 g DM according to WHO’s standards [11]. The arithmetic mean was obtained for 94.8% and 100% of samples except for *Strongyloides* eggs (83.6%).

During the experimental period, clear fluctuations in the mean concentration of protozoan cysts in dried sludge samples could be observed (Figure 6). The number averaged 2.9 cysts/100 g DM for all detected species during the three-year study period. Dried sludge samples contained *Giardia* spp. cysts with a mean concentration of 2.25 ± 0.8 cysts/100 g DM (minimum 0.3 and maximum 4.65/100 g DM). The concentration of *Entamoeba histolytica*/*dispar*/*moshkovskii* and *Entamoeba coli* cysts ranged from 1.8 to 5.8 and 1.3 to 10.7 cysts/100 g DM, with a mean of 1.8 ± 0.8 and 4.7 ± 1.2 cysts/100 g DM, respectively. Based on the results, a relatively strong persistence of the protozoan cysts in sewage sludge was noted. Ben Ayed et al. [63] investigated protozoan molecular analysis in four dried and eight dehydrated sludge samples collected between 2005 and 2008 from 18 wastewater treatment plants located throughout Tunisia. The PCR results identified *Enterocytozoon bieneusi*, *Giardia duodenalis*, and *Cryptosporidium* spp. in one positive sample among the 8 analyzed dried sludge samples (12.5%) and *Eimeria* spp. in 4 positive samples (50%), collected from several plants throughout Tunisia. As stated in the above study, neither *Cryptosporidium* oocysts (4–6 µm) nor other sub-cited species, were detected in any of the dried sludge samples based on Sheather’s sucrose flotation technique. However, they may have remained undetected by the flotation technique applied owing to their size as the major obstacle in their detection and the need to perform specific staining techniques such as Ziehl–Neelsen staining that was found to be efficient in the detection of *Cryptosporidium* oocysts by microscopy [64,65]. Searcy et al. [66] found that the low hydrophobicity and negative charge of *Cryptosporidium* oocysts can be increased by suspensions with high conductivity, which can influence adhesion to hydrophobic surfaces, consequently preventing their adhesion to the sediment and decreasing their removal by sedimentation.

### 3.2. Parasitological Results Analysis of Solar Drying Sludge in Beds

Previous studies of the investigated WWTPs have shown that the sewage sludge before application of solar bed drying, may contain a mean concentration of helminth eggs from 9.6 × 10^1^ to 3.4 × 10^2^ eggs/100 g of sewage sludge, (in outlet of thickeners; data not shown). This provides a general idea about the efficiency of sludge treatment. However, the main goal of this study was not to study the efficiency but rather the quality of the final product used in part, for agricultural land application after solar drying in beds. Thus, the obtained results show that the solar drying process provides low or no residual helminth egg concentrations compared to those obtained at the outlet of thickeners (57.3% of samples with concentration ≤1 egg/100 g DM), and allows total elimination of helminth ova in dried material (approximately 40.8% of samples). However, an unsatisfactory degree of inactivation of the observed intestinal parasites was obtained, limiting the use of dried sludge. In fact, the 116 dried sludge samples showed a portion of 1.7% of samples presenting concentrations higher than 1 per 100 g DM (mix of all main helminth species). Thus, the low mean of intestinal parasites might be due to the dry and warm climate providing better conditions for a good rate of removal efficiency of these parasites, but remains inadequate. In the current study, this may be due to the characteristics of the treatment plants (aerobic digesters not fully functional due to operational and technical problems) and the size of WWTPs demonstrating satisfactory solar drying effects in small and medium sized WWTPs, resulting in fluctuations in the protozoan cyst parasite concentration. Nevertheless, in addition to the physical dewatering performance, solar drying beds provide 15 to 20% of stabilization of thickened sludge. Countermeasures to the technical and financial problems of digestion problems in Tunisian WWTPs may be to increase the use of lime.

Yashas and Udayashankara [67] studied two WWTPs and found that the average (oo) cysts (*Cryptosporidium* spp. and *Giardia* spp.) present in sludge after stabilization (aerobic digestion) was higher than in raw sludge. Moreover, the same authors reported that limited information is available on the fate of protozoan pathogens in biosolids, and that interpretation and comparison of data are difficult due to inconsistencies in sampling, concentration, and recovery procedures.

Concentrations and reduction rates of enteric parasites and other pathogens identified and quantified from dried sewage sludge for various counting methods and different country conditions reported in the literature are promising. Cofie et al. [68] reported that sewage sludge drying beds retained 100% of helminth eggs. Al-Malack [69] showed that sludge samples collected from six different sludge depths (10, 15, 20, 25, 30, and 35 cm) were free from *A. lumbricoides*, *Trichuris* spp., *E. vermicularis*, *H. nana*, and *E. histolytica* after 30 days of bed drying for all sludge depths. Mihelcic [70] highlighted that pathogens in solar drying beds were reduced according to fecal coliforms (1 to 2 log10 reduction), bacteria (2 log10) and viruses (1 log10). However, studies are scant for other pathogens. Microorganisms are extremely sensitive to loss of moisture, implying that the drying of sludge reduces their number [71]. According to Sypuła et al. [72], the agricultural use of sewage sludge is feasible and one of the most efficient methods for treatment is solar drying. Phiri et al. [73] argued that the technology of covered drying beds is relatively simple, cheap, and affordable and can be used year round to treat biosolids. They suggested that further reduction can be achieved with prolonged exposure of the sludge even under rainy ambient conditions provided that water is not affecting the sludge.

The obtained results are inconsistent with the assumption that solar dried sewage sludge in beds are free from parasites. This is likely due to treatment efficiency, investigation methods, and analytical methods not being standardized. According to Jaromin-Gleń et al. [54], there is at present no standard method for the determination of helminth eggs in sewage sludge. In most cases, the standardized flotation method by Spindler is employed, as modified by Wasilkowa, designed mainly for the examination of soil, or the flotation method by Quinn et al. [74]. Numerous authors have recommended the use of flotation solutions with a specific gravity from 1.2 to 1.3. These specific gravities appear to be optimal to ensure the recovery of most eggs and particularly heavier eggs (specific gravity of 1.30) together with protozoan (oo) cysts [62,75,76].

The sucrose flotation method applied in the present study is an efficient technique. It is fast and precise, which facilitates the elimination or considerable limitation of the effect of debris on the effectiveness of the examination. Sucrose flotation technique is generally used for separating organisms based on their specific gravity, it is effective but requires skilled personnel. However, we were unable to show the exact recovery rate of the method for counting of helminth eggs and protozoan cysts. Our methodology was applied to recover these enteric parasites naturally found in field samples (dried sludge samples as final product), without inoculating known concentration of eggs, and cysts of any species. Thus, further studies are needed by seeding dried sludge samples with known concentrations of parasites to determine the accuracy, and recovery rate. Therefore, more research is needed to consider the helminth egg, and protozoan cyst recovery rates from different sets of sludge samples. According to the authors’ knowledge, parasitological studies of dried sewage sludge in Tunisian wastewater treatment plants are scarce or have employed other detection methods such as the modified Bailenger method, immunomagnetic separation followed by immunofluorescent assay microscopy, and PCR [41,43,63].

If the solar drying of sludge in beds is not performed in a satisfactory manner, as noted for 1.7% of dried sludge samples with helminth egg concentrations larger than 1 egg/100 g DM, there are needs for improvement of the operation by adding processes to significantly reduce pathogens such as intestinal parasites through liming or co-composting steps that have shown effectiveness of helminth egg reduction [36,77,78]. In the Tunisian context and for improved treatment, simple, operational, low consumption of energy, and low level of investment should be applied for sewage sludge stabilization allowing for extended time of dewatering on solar drying beds especially during unfavorable conditions such as wet weather and low temperatures. Solar energy is a viable alternative for developing countries such as Tunisia. Solar energy is renewable and can be used to efficiently treat sludge in a sustainable manner. The efficiency of solar drying in beds, however, entirely depends on climatic conditions in particular solar radiation and temperature. In this respect, the Tunisian climate provides one the most favorable conditions worldwide by receiving more than 3000 h of sun per year corresponding to 11.6 ± 0.2 h per day resulting in decreased moisture concentration and consequently pathogen reduction. As reported by Seginer and Bux [79] and Shanahan et al. [80], solar drying is a sustainable and efficient method to disinfect and stabilize sewage sludge. Based on the present findings, we seek to further investigate the behavior of pathogens in drying beds depending on time since filling, combined with other potential risk estimations to determine an adequate retention time for maximum reduction of intestinal parasites.

For such an evaluation, it would be necessary to prove the viability of the parasites and achieve a quantitative microbiological risk assessment for public health. However, the morphological characteristics indicated complete intactness of eggs and cysts.

The number of eggs detected in the samples did not exceed the allowable standards established by the international regulations previously described such as the French Decree of 8 January [12] or category A sludge according to American CFR Part 503 regulation [81], and World Health Organization [11]. In the present study, we focused mainly on detecting the presence of enteric parasites in dried sludge samples as a final product reused in agriculture as fertilizer. The Tunisian standard NT 106.20 [32] that is currently in place does not have limit values set for pathogenic helminth eggs and protozoan cysts. Studies are needed to create limits for these intestinal parasites in sludge suitable for application to agricultural land. With such tools, it will become possible to prepare the sludge for reuse in agriculture with greater control as a first step of a risk assessment process by hazard identification. The results of this study can be compared to other regions with a similar climate and where similar treatment methods are used. 

## 4. Conclusions

The saturated sucrose flotation technique was successfully used to evaluate the majority of helminth eggs and protozoan cysts (mainly *Giardia*, *Entamoeba histolytica*/*moshkovski*/*dispar*, and *Entamoeba coli*) in sun-dried sludge samples. The saturated sucrose flotation solution used with a specific gravity of 1.29 was effective in recovering eggs of different species of helminths mainly *Strongyloides* spp., Taeniid eggs, *Strongyloides stercoralis*, *Hymenolepis nana*, *Ascaris* spp., *Enterobius vermicularis*, hookworm species and rhabditoid larvae.

Due to the low concentration of intestinal parasites in the dried sludge, it is essential to develop and evaluate complementary laboratory concentration methods for the recovery, detection, and identification of the pathogens and to increase the quality of results.

Considering the WHO and US EPA standard limit of helminth eggs in sludge intended for agricultural use, mean concentration during the three years of the investigation period was less than 1 egg/100 g DM.

Sewage sludge solar drying in beds, thus, appears to be a technology that guarantees obtaining biologically safe material for agricultural purposes according to international standards mentioned above. As a first diagnosis and upfront-study, our evaluation of enteric parasites was based mainly on morphological characteristics when observed under microscopy indicating complete integrity of helminth eggs and protozoan cysts. However, in order to assess true parasitological safety, further studies are needed in our future investigations considering the determination of the viability of these parasite eggs isolated from dried sewage sludge.

## Figures and Tables

**Table 1:** Standards for maximum concentration of intestinal parasites for sewage sludge use in agriculture.

Country	Intestinal Parasites	Reference
WHO	<1 viable helminth egg/g DM	[11]
France	<3 viable helminth egg/10 g DM	[12]
Poland	0 live egg of intestinal parasites (*Ascaris* spp., *Trichuris* spp., *Toxocara* spp.)/kg DM	[13,14,15,16]
Lithuania	Helminth egg and larvae, 0 units/kg	[13,14]
Luxembourg	No eggs of worm likely be contagious	[13,14]
Bulgaria	Viable helminth egg and larvae, 1 unit/kg DM	[13,14,17]
Austria (Carinthia)	No helminth egg (Applied to all classes)	[14]
Austria (Lower Austria)	No helminth egg	[14]
Austria (Steiermark)	No helminth egg	[14]
Norway	0 helminth egg/g TS (Dry weight basis)	[18]
Brazil	0.25 viable helminth egg/g DM (Class A) 10 viable helminth eggs/g DM (Class B)	[19]
Chile	0.25 helminth egg/g DM (Class A)	[20]
Mexico	<1 viable helminth egg/g DM (Class A) <10 helminth eggs/g DM (Class B)	[21]
New Zealand	<1 Helminth egg/4 g TS (Class A)	[22,23]
South Africa	1 viable helminth egg/g DM (Class A) 4 viable helminth eggs/g DM (Class B) >4 viable helminth eggs/g DM (Class C)	[24]
United States	<1 viable helminth egg/4 g TS (dry weight basis)	[25]
Colombia	<1 viable helminth egg/4 g DM (Class A) <10 viable helminth eggs/4 g DM (Class B)	[14,26]
Russia	<1 Viable eggs of geohelminths (roundworms, whipworms, hookworms)/1 dm^3^ DM <1 Viable eggs of biohelminths (oncospheres of teniid, eggs of fascioli)/1 dm^3^ DM <1 viable cysts of intestinal pathogenic protozoa (cysts of *Giardia*, *Balantidium*, *Cryptosporidium* oocysts/1 dm^3^ DM	[27]
Jordan	<1 worm live ova/4 g DM (Sludge treated to the second level)	[28]

**Table 2:** Main characteristics of sewage sludge from WWTPs used in this study [5].

Design and Performance									Characteristics of Dried Sludge			
Plant	District	Capacity EI	Flow Rate (m^3^/Day)	kg BOD_5_/Day	Secondary Wastewater Treatment	Treatment Efficiency (%)			Treatment Type	Volume (m^3^)	Dry Matter (%)	Coordinates
						BOD_5_	COD	TSS				
WWTP1	DT	51,000	2800	1704	OD	96	93	91	T+DB	431	77	36°51′47.62″ N; 9°57′10.60″ E
WWTP2	NW	17,968	1280	719	OD	91	86	82	AD+T+DB	292	75	36°27′50.46″ N; 9°16′8.57″ E
WWTP3	NW	18,874	1180	720	OD	93	90	91	AD+T+DB	180	64	36°34′0.18″ N; 9°26′35.41″ E
WWTP4	NW	51,000	4530	2450	OD	80	76	68	T+DB	750	80–90	36°7′11.09″ N; 9°23′0.75″ E
WWTP5	NW	95,000	8500	4000	LLAS	91	90	91	T+DB	986	74–90	36°8′19.63″ N; 8°41′6.07″ E
WWTP6	C	236,000	20,000	9000	OD	94	93	94	T+DB	5768	49–65	35°43′46.59″ N; 10°6′53.86″ E
WWTP7	SE	19,500	1395	700	OD	97	94	97	T+DB	-	90	36°40′29.71″ N; 10°32′58.68″ E
WWTP8	E	17,000	1500	600	OD	95	92	95	T+DB	98	50–80	35°43′13.54″ N; 10°40′26.87″ E
WWTP9	SE	526,800	49,500	21,600	OD	79	71	72	T+DB	2790	84	34°50′2.13″ N; 10°51′15.70″ E
WWTP10	E	10,000	780	400	OD	87	81	86	DB	362	86	34°31′3.80″ N; 10°29′34.66″ E

WWTP: Wastewater treatment plant; DT: District of Tunis; NW: Northwest; C: Center; SE: South East; E: East; EI: Equivalent Inhabitant; BOD_5_: Biochemical Oxygen Demand over 5 days; COD: Chemical Oxygen Demand; TSS: Total Suspended Solids; OD: Oxidation Ditches; AD: Aerobic Digestion; T: Thickening; DB: Drying Beds; LLAS: Low Load Activated Sludge; -: Data not available.

**Table 3:** Descriptive statistics of Helminth ova and protozoan cysts obtained in the dried sludge samples per 100 g DM (N = 116) for the 10 investigated WWTPs during 2013–2015.

	Helmith Eggs											Protozoan Cysts		
	Nematodes								Cestodes			Flagellates	Amoebas	
Parasite	*Asc*. spp.	*Toxo*. spp.	*E. v.*	*Trich*.spp.	*H. W*	*S. s.*	*Strg*. spp.	*Tris*. spp.	Tae eggs	*H. d.*	*H. n.*	*G.* spp.	*E. h.*	*Entamoeba coli*
Min	0.00	0.00	0.00	0.00	0.00	0.00	0.00	0.00	0.00	0.00	0.00	0.33	0.33	1.32
1st. Qu.	0.00	0.00	0.00	0.00	-	-	0.33	0.00	0.00	-	-	1.65	1.32	3.99
Medium	0.33	0.00	0.00	0.00	0.00	0.33	0.66	0.00	0.33	0.00	0.33	2.31	1.66	4.65
Mean *	0.28	≅0.00 **	0.14	≅0.00 **	0.08	0.32	0.6	0.15	0.31	≅0.00 **	0.25	2.25	1.79	4.74
3rd. Qu.	0.33	0.00	0.33	0.00	0.00	0.33	0.66	0.33	0.33	0.00	0.33	2.67	2.32	5.64
Max.	1.78	0.33	1.33	0.33	0.66	1.10	3.30	0.66	1.98	0.33	0.89	4.65	5.78	10.7
Std. Dev.	0.34	0.05	0.21	0.03	0.15	0.27	0.42	0.20	0.31	0.04	0.23	0.81	0.82	1.23
C.V.	1.21	5.0	1.50	-	1.87	0.84	0.7	1.33	1.03	-	0.92	0.36	0.46	0.26
Sum	32.4	0.82	14.4	0.33	8.52	35.9	70.14	16.5	37.76	0.66	29.1	260.6	210.1	538.1
% positive	56.9	2.6	37.9	0.9	28.45	72.4	91.4	44.0	74.1	1.72	67.2	100.0	100.0	100.0

Mean *: arithmetic mean; **: low arithmetic mean concentration close to zero: They were not detected in the majority of the dried sludge samples compared to eggs of remaining detected species; Max.: maximum; Min.: minimum; Std. Dev.: Standard deviation; C.V.: Coefficient of variation; Sum: Total helminthes; *Asc*. spp.: *Ascaris* spp; *Toxo*. spp.: *Toxocara* spp.; E.v.: *Enterobius vermicularis*; Trich. spp.: *Trichuris* spp.; H. W: Hookworms (*Ancylostoma duodenale* and *Necator americanus*); S. s.: *Strongyloides stercoralis*; *Strg*. spp.: *Strongyloides* spp.; *Tris*. spp.: *Tristrongyloides* spp.; Tae eggs: Taeniid eggs; *H. d.*: *Hymenolepis diminuta*; *H. n.*: *Hymenolepis nana*; *G*. spp.: *Giardia* spp.; *E. h.*: *Entamoeba histolytica*/*dispar*/*moshkovskii*.

## Data Availability

Not applicable.
